# Supplementary material for: A Likelihood Approach for Real-Time Calibration of Stochastic Compartmental Epidemic Models
Source: PLoS Comput Biol. 2017 Jan 17;13(1):e1005257. doi: 10.1371/journal.pcbi.1005257 (PMC5240920; doi:10.1371/journal.pcbi.1005257)
Supplement: S1 File — (TAR.GZ) [file pcbi.1005257.s014.tar.gz › HSPH_Online-SI-Revision/IPoi09/n1-severe/n1-severe_BENCH2_table0.pdf]

1	newly 3 weeks spec	27.	41.7321	{22., 63.}	63.955	0.203302	newly 3 weeks cumul	92.	143.696	{82., 201.}	62.2089	0.199465	newly 5 weeks spec	28.	27.0893	{12., 47.}	38.4566	0.187748	newly 5 weeks cumul	149.	205.339	{108., 290.}	48.9573	0.170549	newly 54 weeks cumul	407.	315.179	{149., 506.}	33.231	0.193913
2	newly 3 weeks spec	51.	46.1268	{23., 74.}	32.3667	0.165072	newly 3 weeks cumul	151.	160.93	{101., 223.}	27.115	0.115659	newly 5 weeks spec	43.	34.3521	{14., 59.}	40.4193	0.234934	newly 5 weeks cumul	237.	235.155	{139., 354.}	28.4483	0.131123	newly 56 weeks cumul	398.	369.408	{202., 604.}	34.369	0.167652
3	newly 3 weeks spec	41.	55.7705	{36., 76.}	45.7817	0.167574	newly 3 weeks cumul	126.	178.738	{126., 223.}	45.8886	0.16083	newly 5 weeks spec	34.	42.6557	{23., 60.}	40.1157	0.151081	newly 5 weeks cumul	205.	269.443	{178., 336.}	38.5206	0.142489	newly 55 weeks cumul	432.	459.361	{276., 641.}	27.9637	0.122997
4	newly 3 weeks spec	70.	75.6761	{57., 99.}	21.992	0.0972827	newly 3 weeks cumul	194.	241.507	{197., 286.}	31.4288	0.129893	newly 5 weeks spec	40.	57.7887	{33., 81.}	51.2324	0.171344	newly 5 weeks cumul	270.	366.521	{298., 435.}	40.5373	0.151103	newly 58 weeks cumul	393.	604.831	{432., 780.}	54.6895	0.179898
5	newly 3 weeks spec	50.	68.5672	{49., 84.}	39.6418	0.138925	newly 3 weeks cumul	135.	230.866	{187., 274.}	71.0116	0.227877	newly 5 weeks spec	19.	48.4328	{31., 68.}	154.91	0.383199	newly 5 weeks cumul	204.	337.985	{249., 409.}	65.679	0.212688	newly 57 weeks cumul	333.	510.164	{341., 687.}	53.8658	0.175764
6	newly 3 weeks spec	37.	48.2364	{28., 64.}	39.9017	0.149689	newly 3 weeks cumul	122.	147.509	{77., 185.}	33.9344	0.137796	newly 5 weeks spec	35.	44.5818	{31., 63.}	34.961	0.125616	newly 5 weeks cumul	196.	240.036	{154., 298.}	31.2616	0.122711	newly 54 weeks cumul	501.	556.255	{392., 703.}	23.1646	0.0938767
7	newly 3 weeks spec	30.	35.0508	{17., 53.}	38.7571	0.157518	newly 3 weeks cumul	90.	128.102	{65., 183.}	51.7514	0.179476	newly 5 weeks spec	29.	24.8136	{11., 39.}	37.5804	0.200763	newly 5 weeks cumul	156.	183.39	{93., 269.}	34.3981	0.138472	newly 54 weeks cumul	252.	289.695	{140., 431.}	38.5526	0.154396
8	newly 3 weeks spec	38.	78.2466	{53., 103.}	106.273	0.299489	newly 3 weeks cumul	207.	254.658	{198., 311.}	26.2656	0.0993863	newly 5 weeks spec	30.	52.6438	{30., 78.}	77.8539	0.229528	newly 5 weeks cumul	276.	371.863	{277., 451.}	37.433	0.134114	newly 58 weeks cumul	301.	546.836	{365., 717.}	82.119	0.246034
9	newly 3 weeks spec	23.	50.7705	{33., 68.}	125.303	0.371592	newly 3 weeks cumul	75.	159.623	{114., 212.}	116.94	0.33917	newly 5 weeks spec	38.	41.5738	{23., 59.}	33.3046	0.148175	newly 5 weeks cumul	137.	246.246	{170., 308.}	84.6955	0.267961	newly 54 weeks cumul	335.	473.279	{289., 637.}	49.8116	0.175977
10	newly 3 weeks spec	42.	52.0588	{29., 76.}	37.7451	0.144926	newly 3 weeks cumul	146.	171.5	{106., 230.}	30.701	0.125569	newly 5 weeks spec	28.	38.9265	{19., 65.}	54.5693	0.180829	newly 5 weeks cumul	205.	254.971	{152., 356.}	35.6958	0.133712	newly 54 weeks cumul	314.	434.25	{241., 689.}	49.7705	0.166485
11	newly 3 weeks spec	26.	37.55	{21., 54.}	53.0128	0.178678	newly 3 weeks cumul	86.	118.533	{81., 148.}	42.2868	0.14769	newly 5 weeks spec	24.	31.95	{16., 52.}	49.5139	0.173735	newly 5 weeks cumul	140.	186.883	{120., 248.}	39.0119	0.138386	newly 51 weeks cumul	390.	389.25	{227., 633.}	36.6026	0.164093
12	newly 3 weeks spec	37.	69.3662	{46., 105.}	87.7807	0.254539	newly 3 weeks cumul	137.	254.437	{187., 307.}	85.7202	0.26042	newly 5 weeks spec	21.	41.6479	{21., 67.}	100.469	0.263857	newly 5 weeks cumul	184.	350.62	{243., 471.}	90.5542	0.267905	newly 59 weeks cumul	237.	460.352	{290., 661.}	94.2533	0.269073
13	newly 3 weeks spec	52.	50.9194	{33., 72.}	26.8921	0.157172	newly 3 weeks cumul	157.	164.194	{110., 212.}	25.0051	0.142385	newly 5 weeks spec	31.	38.4032	{16., 64.}	47.9188	0.214242	newly 5 weeks cumul	230.	247.355	{145., 340.}	29.1304	0.157523	newly 54 weeks cumul	413.	432.694	{225., 674.}	32.7853	0.173042
14	newly 3 weeks spec	54.	80.4219	{57., 104.}	54.3113	0.197358	newly 3 weeks cumul	204.	265.422	{207., 318.}	35.7613	0.153081	newly 5 weeks spec	35.	59.3281	{34., 84.}	73.7054	0.228715	newly 5 weeks cumul	282.	397.188	{333., 478.}	45.9774	0.17716	newly 59 weeks cumul	417.	620.844	{439., 780.}	49.7752	0.167357
15	newly 3 weeks spec	15.	31.7813	{14., 53.}	116.667	0.299751	newly 3 weeks cumul	83.	114.391	{63., 176.}	49.7553	0.168743	newly 5 weeks spec	28.	21.8906	{8., 43.}	48.4933	0.28809	newly 5 weeks cumul	134.	161.984	{84., 256.}	42.0126	0.159602	newly 53 weeks cumul	180.	249.891	{112., 431.}	56.033	0.180639
16	newly 3 weeks spec	53.	73.7143	{47., 96.}	45.4328	0.15842	newly 3 weeks cumul	165.	242.825	{169., 297.}	48.4175	0.165779	newly 5 weeks spec	40.	52.9365	{32., 91.}	47.3413	0.16643	newly 5 weeks cumul	245.	358.238	{259., 438.}	48.3706	0.166317	newly 59 weeks cumul	406.	532.079	{356., 785.}	38.0835	0.133867
17	newly 3 weeks spec	52.	71.3472	{43., 105.}	45.2724	0.153981	newly 3 weeks cumul	182.	257.611	{190., 332.}	43.6661	0.149701	newly 5 weeks spec	22.	40.1389	{21., 64.}	86.7424	0.239331	newly 5 weeks cumul	246.	355.056	{246., 489.}	47.0754	0.157741	newly 59 weeks cumul	311.	467.833	{315., 676.}	53.921	0.173945
18	newly 3 weeks spec	25.	69.6	{33., 97.}	186.4	0.45602	newly 3 weeks cumul	80.	213.183	{107., 283.}	174.813	0.44824	newly 5 weeks spec	24.	56.1333	{24., 85.}	138.333	0.351247	newly 5 weeks cumul	126.	332.5	{166., 424.}	169.365	0.424197	newly 59 weeks cumul	490.	565.367	{354., 738.}	29.2653	0.122079
19	newly 3 weeks spec	33.	38.0758	{18., 67.}	43.1129	0.20038	newly 3 weeks cumul	111.	129.409	{73., 191.}	36.2954	0.16522	newly 5 weeks spec	35.	26.6212	{12., 50.}	46.5368	0.289603	newly 5 weeks cumul	180.	188.045	{103., 294.}	33.9141	0.171923	newly 52 weeks cumul	331.	301.97	{146., 548.}	38.6798	0.216503
20	newly 3 weeks spec	32.	40.5373	{19., 64.}	48.8806	0.216144	newly 3 weeks cumul	124.	138.612	{72., 206.}	36.7959	0.183692	newly 5 weeks spec	27.	29.2985	{10., 51.}	45.3289	0.253258	newly 5 weeks cumul	188.	203.	{104., 305.}	35.9082	0.186881	newly 52 weeks cumul	258.	344.179	{163., 608.}	54.3446	0.212143
21	newly 3 weeks spec	34.	31.3273	{11., 64.}	50.8556	0.263673	newly 3 weeks cumul	127.	125.836	{45., 232.}	44.6099	0.216486	newly 5 weeks spec	35.	16.5455	{3., 33.}	66.8571	0.557524	newly 5 weeks cumul	201.	165.327	{53., 303.}	43.365	0.246787	newly 59 weeks cumul	389.	210.2	{55., 437.}	55.2279	0.403155
22	newly 3 weeks spec	28.	48.3134	{24., 75.}	76.8124	0.223507	newly 3 weeks cumul	132.	159.552	{105., 215.}	32.9489	0.125936	newly 5 weeks spec	29.	35.8955	{16., 63.}	52.5991	0.199224	newly 5 weeks cumul	187.	237.91	{149., 346.}	38.1435	0.137625	newly 53 weeks cumul	239.	406.179	{198., 694.}	76.8188	0.221401
23	newly 3 weeks spec	28.	33.3462	{10., 54.}	51.6484	0.22821	newly 3 weeks cumul	106.	124.981	{55., 180.}	41.3824	0.175338	newly 5 weeks spec	31.	20.4423	{7., 35.}	44.727	0.310418	newly 5 weeks cumul	167.	172.423	{72., 255.}	34.4081	0.167266	newly 55 weeks cumul	324.	241.	{95., 364.}	35.2445	0.221201
24	newly 3 weeks spec	36.	24.5538	{10., 41.}	41.8803	0.262804	newly 3 weeks cumul	101.	91.5385	{50., 139.}	31.3328	0.156869	newly 5 weeks spec	23.	18.1385	{4., 33.}	54.4482	0.331546	newly 5 weeks cumul	158.	131.277	{63., 230.}	36.1733	0.192697	newly 52 weeks cumul	238.	212.708	{89., 359.}	47.1493	0.229966
25	newly 3 weeks spec	53.	36.9286	{19., 59.}	35.1752	0.217069	newly 3 weeks cumul	164.	122.625	{77., 178.}	28.9961	0.172744	newly 5 weeks spec	31.	27.9464	{12., 48.}	39.5737	0.206679	newly 5 weeks cumul	226.	182.357	{104., 271.}	28.9981	0.166416	newly 50 weeks cumul	295.	343.607	{158., 633.}	45.0121	0.175823
26	newly 3 weeks spec	42.	40.5079	{21., 67.}	34.6939	0.167518	newly 3 weeks cumul	125.	146.968	{83., 209.}	33.9556	0.130322	newly 5 weeks spec	18.	26.4603	{10., 53.}	72.7513	0.232705	newly 5 weeks cumul	181.	205.46	{110., 311.}	34.1928	0.136813	newly 57 weeks cumul	332.	295.143	{132., 486.}	38.3917	0.19123
27	newly 3 weeks spec	52.	61.8125	{32., 92.}	37.5601	0.146533	newly 3 weeks cumul	169.	199.469	{138., 269.}	30.3809	0.12105	newly 5 weeks spec	14.	47.1094	{25., 80.}	237.165	0.480907	newly 5 weeks cumul	215.	300.609	{197., 410.}	45.1526	0.154081	newly 56 weeks cumul	229.	490.094	{280., 731.}	114.902	0.304577
28	newly 3 weeks spec	51.	79.2464	{53., 102.}	57.2606	0.187673	newly 3 weeks cumul	140.	249.101	{197., 297.}	78.3437	0.245944	newly 5 weeks spec	48.	59.0435	{30., 89.}	36.8961	0.138861	newly 5 weeks cumul	238.	378.203	{278., 443.}	59.0793	0.195388	newly 59 weeks cumul	446.	587.797	{370., 775.}	37.6812	0.134757
29	newly 3 weeks spec	34.	27.5254	{12., 50.}	39.9801	0.22865	newly 3 weeks cumul	114.	101.	{48., 164.}	34.0321	0.17812	newly 5 weeks spec	17.	19.1186	{9., 34.}	45.9621	0.18801	newly 5 weeks cumul	151.	142.	{65., 238.}	35.9075	0.176688	newly 52 weeks cumul	284.	215.407	{97., 362.}	38.8577	0.226942
30	newly 3 weeks spec	37.	39.0571	{22., 72.}	36.4479	0.15413	newly 3 weeks cumul	120.	135.686	{83., 216.}	31.0476	0.123274	newly 5 weeks spec	28.	26.3	{12., 49.}	39.3367	0.19094	newly 5 weeks cumul	177.	193.414	{114., 316.}	31.2268	0.126057	newly 53 weeks cumul	261.	303.614	{162., 550.}	41.0673	0.153688
31	newly 3 weeks spec	31.	29.4821	{9., 58.}	48.3295	0.259444	newly 3 weeks cumul	87.	102.839	{30., 191.}	49.5279	0.205947	newly 5 weeks spec	16.	20.2679	{4., 45.}	73.1027	0.348426	newly 5 weeks cumul	126.	148.	{44., 259.}	51.4739	0.218459	newly 54 weeks cumul	328.	231.143	{47., 515.}	52.4608	0.348067
32	newly 3 weeks spec	17.	59.7333	{39., 80.}	257.059	0.551045	newly 3 weeks cumul	59.	174.633	{101., 225.}	203.107	0.489205	newly 5 weeks spec	26.	51.5833	{32., 72.}	104.038	0.311763	newly 5 weeks cumul	103.	284.283	{197., 354.}	181.44	0.454886	newly 56 weeks cumul	357.	601.	{404., 761.}	68.3473	0.215496
33	newly 3 weeks spec	51.	78.8529	{49., 109.}	61.5917	0.208603	newly 3 weeks cumul	172.	261.529	{198., 328.}	57.2845	0.204021	newly 5 weeks spec	50.	52.4265	{30., 87.}	34.3235	0.152361	newly 5 weeks cumul	284.	379.074	{278., 486.}	39.7732	0.150206	newly 59 weeks cumul	418.	558.132	{367., 796.}	39.6461	0.138095
34	newly 3 weeks spec	13.	17.	{3., 39.}	76.4016	0.30491	newly 3 weeks cumul	56.	63.1356	{15., 127.}	62.0763	0.282029	newly 5 weeks spec	29.	9.33898	{2., 20.}	69.6669	0.700988	newly 5 weeks cumul	105.	85.8305	{19., 162.}	51.251	0.318038	newly 51 weeks cumul	255.	116.	{23., 220.}	59.7474	0.507988
35	newly 3 weeks spec	56.	79.8696	{51., 102.}	46.765	0.161255	newly 3 weeks cumul	196.	267.29	{196., 323.}	37.6442	0.133951	newly 5 weeks spec	34.	54.6087	{31., 86.}	63.7681	0.191547	newly 5 weeks cumul	269.	389.551	{293., 465.}	45.5363	0.157444	newly 59 weeks cumul	333.	558.522	{378., 788.}	68.2291	0.212556
36	newly 3 weeks spec	35.	35.1714	{14., 61.}	39.3469	0.181433	newly 3 weeks cumul	137.	177.686	{85., 282.}	45.3597	0.161594	newly 5 weeks spec	9.	15.0429	{5., 29.}	87.1429	0.266849	newly 5 weeks cumul	166.	215.643	{108., 335.}	46.3769	0.164985	newly 60 weeks cumul	168.	238.857	{116., 369.}	56.0034	0.186225
37	newly 3 weeks spec	30.	29.0317	{15., 49.}	35.1852	0.166082	newly 3 weeks cumul	103.	104.556	{57., 161.}	31.931	0.143769	newly 5 weeks spec	13.	22.5556	{10., 43.}	84.9817	0.242095	newly 5 weeks cumul	136.	151.222	{84., 252.}	36.2628	0.14813	newly 50 weeks cumul	294.	242.762	{120., 429.}	36.8643	0.197295
38	newly 3 weeks spec	36.	69.5	{45., 96.}	93.3642	0.270609	newly 3 weeks cumul	146.	244.944	{182., 302.}	67.9985	0.2177	newly 5 weeks spec	22.	43.7361	{25., 64.}	100.063	0.273714	newly 5 weeks cumul	199.	344.264	{246., 459.}	73.2901	0.228266	newly 58 weeks cumul	228.	477.653	{320., 690.}	109.497	0.304371
39	newly 3 weeks spec	27.	53.9265	{34., 72.}	103.649	0.311615	newly 3 weeks cumul	108.	172.544	{117., 216.}	64.0387	0.232756	newly 5 weeks spec	28.	41.1912	{25., 57.}	55.0945	0.208432	newly 5 weeks cumul	158.	261.735	{169., 328.}	69.3038	0.241611	newly 54 weeks cumul	281.	450.279	{269., 609.}	65.4647	0.230979
40	newly 3 weeks spec	44.	38.3793	{7., 85.}	53.7618	0.349662	newly 3 weeks cumul	123.	173.345	{51., 345.}	66.2461	0.231762	newly 5 weeks spec	23.	17.2414	{3., 43.}	67.0165	0.506062	newly 5 weeks cumul	179.	215.724	{55., 416.}	58.3317	0.239516	newly 60 weeks cumul	274.	243.931	{60., 518.}	50.8054	0.276548
41	newly 3 weeks spec	39.	67.1333	{42., 97.}	73.9145	0.222188	newly 3 weeks cumul	166.	229.853	{173., 287.}	40.0884	0.140535	newly 5 weeks spec	19.	44.44	{24., 81.}	134.035	0.328752	newly 5 weeks cumul	210.	329.6	{239., 434.}	57.8794	0.187572	newly 57 weeks cumul	257.	480.2	{321., 710.}	87.4189	0.252139
42	newly 3 weeks spec	38.	41.7885	{17., 66.}	40.6377	0.179392	newly 3 weeks cumul	107.	156.135	{84., 225.}	57.8181	0.201007	newly 5 weeks spec	31.	24.6538	{9., 48.}	46.402	0.281852	newly 5 weeks cumul	165.	214.404	{111., 329.}	48.1235	0.183351	newly 57 weeks cumul	391.	284.923	{126., 456.}	36.6614	0.223652
43	newly 3 weeks spec	32.	35.7742	{19., 55.}	36.4919	0.150531	newly 3 weeks cumul	103.	123.065	{78., 167.}	31.9449	0.121575	newly 5 weeks spec	12.	28.7742	{13., 46.}	143.548	0.356422	newly 5 weeks cumul	138.	182.935	{106., 263.}	41.1875	0.145264	newly 50 weeks cumul	246.	332.823	{164., 534.}	50.0721	0.169533
44	newly 3 weeks spec	38.	70.7867	{51., 91.}	88.4561	0.270809	newly 3 weeks cumul	148.	232.053	{190., 286.}	59.2973	0.206056	newly 5 weeks spec	25.	51.04	{30., 83.}	105.973	0.285836	newly 5 weeks cumul	218.	341.947	{269., 424.}	59.2294	0.199545	newly 58 weeks cumul	302.	535.72	{364., 766.}	78.1501	0.235843
45	newly 3 weeks spec	39.	54.5417	{31., 80.}	47.8276	0.16367	newly 3 weeks cumul	137.	193.875	{132., 255.}	45.4075	0.158356	newly 5 weeks spec	24.	40.5833	{22., 65.}	72.9167	0.212363	newly 5 weeks cumul	197.	282.222	{188., 394.}	46.4749	0.156253	newly 55 weeks cumul	330.	435.278	{275., 678.}	40.8081	0.139044
46	newly 3 weeks spec	24.	43.6296	{24., 64.}	86.1111	0.253849	newly 3 weeks cumul	77.	143.093	{90., 203.}	87.5661	0.258375	newly 5 weeks spec	26.	34.037	{15., 56.}	48.5755	0.177361	newly 5 weeks cumul	128.	216.333	{130., 296.}	72.4248	0.224268	newly 54 weeks cumul	420.	396.833	{207., 606.}	31.2831	0.152598
47	newly 3 weeks spec	53.	81.9265	{55., 112.}	56.576	0.181376	newly 3 weeks cumul	225.	293.588	{220., 356.}	32.5098	0.118475	newly 5 weeks spec	15.	48.6618	{26., 82.}	224.412	0.473722	newly 5 weeks cumul	267.	407.956	{293., 515.}	53.3432	0.176988	newly 60 weeks cumul	272.	532.603	{351., 766.}	95.8099	0.276287
48	newly 3 weeks spec	21.	39.4844	{20., 61.}	94.122	0.263665	newly 3 weeks cumul	100.	138.453	{77., 208.}	48.2031	0.168366	newly 5 weeks spec	11.	26.7344	{9., 59.}	150.71	0.343408	newly 5 weeks cumul	125.	198.	{114., 301.}	65.35	0.204994	newly 54 weeks cumul	216.	303.344	{148., 505.}	56.2066	0.181279
49	newly 3 weeks spec	40.	62.3429	{37., 95.}	59.2857	0.185509	newly 3 weeks cumul	144.	221.514	{149., 292.}	55.119	0.179274	newly 5 weeks spec	31.	39.6286	{22., 71.}	41.0138	0.138203	newly 5 weeks cumul	205.	311.5	{208., 450.}	53.8746	0.173637	newly 58 weeks cumul	301.	436.514	{271., 692.}	49.9003	0.161438
50	newly 3 weeks spec	29.	55.6567	{39., 75.}	97.5811	0.324313	newly 3 weeks cumul	126.	178.522	{133., 230.}	48.4838	0.22004	newly 5 weeks spec	18.	43.6716	{23., 62.}	145.771	0.381982	newly 5 weeks cumul	161.	272.299	{198., 345.}	74.9142	0.265825	newly 56 weeks cumul	315.	489.582	{305., 662.}	61.1372	0.223471
